# Cylindromicin from Arctic-Derived Fungus *Tolypocladium* sp. SCSIO 40433

**DOI:** 10.3390/molecules26041080

**Published:** 2021-02-18

**Authors:** Imran Khan, Jing Peng, Zhuangjie Fang, Wei Liu, Wenjun Zhang, Qingbo Zhang, Liang Ma, Guangtao Zhang, Changsheng Zhang, Haibo Zhang

**Affiliations:** 1 Key Laboratory of Tropical Marine Bio-resources and Ecology, Guangdong Key Laboratory of Marine Materia Medica, RNAM Center for Marine Microbiology, South China Sea Institute of Oceanology, Chinese Academy of Sciences, 164 West Xingang Road, Guangzhou 510301, China; imranmb_kust@yahoo.com (I.K.); urnotpengjing@163.com (J.P.); fangzhuangjie16@mails.ucas.ac.cn (Z.F.); liuwei@scsio.ac.cn (W.L.); wzhang@scsio.ac.cn (W.Z.); gudaobo@163.com (Q.Z.); maliangyc@hotmail.com (L.M.); gtzhang@scsio.ac.cn (G.Z.); 2 South China Sea Institute of Oceanology, University of Chinese Academy of Sciences, Beijing 100049, China; 3 Southern Marine Science and Engineering Guangdong Laboratory (Guangzhou), 1119 Haibin Road. Nansha District, Guangzhou 511458, China; 4 Sanya Institute of Oceanology, SCSIO, Sanya 572000, China

**Keywords:** Arctic, fungi, cylindromicin, tyrosinase

## Abstract

The fungus strain SCSIO 40433 was isolated from an Arctic-derived glacier sediment sample and characterized as *Tolypocladium cylindrosporum*. A new compound, cylindromicin (**1**), and seven known secondary metabolites (**2**–**8**) were isolated from this strain. The chemical structures of these compounds were elucidated by comprehensive spectroscopic analyses. Cylindromicin (**1**) featured a 3,4-dihydro-2*H*-pyran skeleton. The absolute configuration of compound **1** was assigned via interpretation of key Nuclear Overhauser Effect Spectroscopy (NOESY) correlations and Electronic Circular Dichroism (ECD) calculation. Cylindromicin (**1**) exhibited significant tyrosinase inhibition activity. This study highlights Polar fungi as a potential resource for new bioactive natural products.

## 1. Introduction

Polar regions are inaccessible and intriguing areas on the Earth because of the aurora, extreme low temperature, and polar day and night cycles [1]. Polar regions consist of the Arctic, the Antarctic, and their sub regions. Diverse groups of microorganisms have been isolated in the polar regions, including bacteria, actinomycetes, and fungi, which can adapt to the extreme environments [2,3,4]. Nowadays, most of the identified and investigated polar microorganisms belong to the Antarctic regions [5,6,7,8,9,10,11,12]. In contrast, only a few Arctic-derived microorganisms have been isolated and screened for secondary metabolites [13]. The Arctic-derived bioactive natural compounds, such as psychrophilin D [3], eutypellenoids A–C [14], eutypellacytosporins A−D [15], libertellenones G–H [16], and libertellenones M–N [17], were isolated from Arctic fungi *Penicillium algidum* and *Eutypella* species. They showed diverse bioactivities, including cytotoxic activities, antifungal and antibacterial activities. Interestingly, some marine bacteria and fungi have been observed to produce tyrosinase inhibitors, which have been investigated for their employment in cosmetic products [18]. Kojic acid is a well-known tyrosinase inhibitor produced by *Aspergillus* and *Penicillium* fungi [19,20], that has been widely used in the cosmetic industries as a skin lightning/depigmenting agent [21]. Due to the metabolic instability of kojic acid, many efforts have been devoted to finding more powerful tyrosinase inhibitors [22,23,24,25]. However, attempts to discover tyrosinase inhibitors from Arctic-derived microbes are rarely found in the literature [26,27].

**Figure 1:**
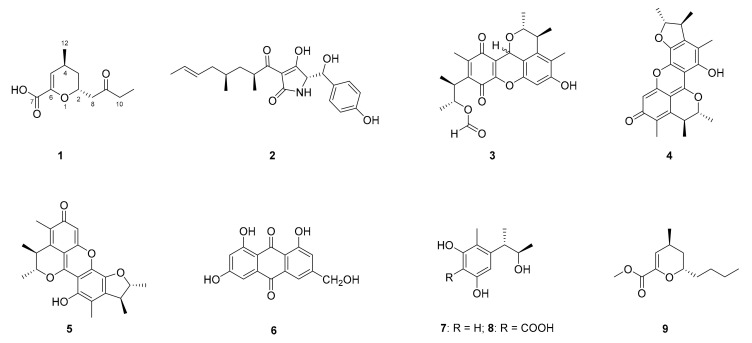
Chemical structures of compounds **1**–**9**.

In this work, we reported the isolation of a fungus strain SCSIO 40433 from the glacier sediment sample and the identification of the strain as *Tolypocladium cylindrosporum*. Our investigations on SCSIO 40433 led to the discovery of a series of secondary metabolites, including a new compound, cylindromicin (**1**), and seven known compounds (**2**–**8**, Figure 1). Cylindromicin (**1**) exhibited significant tyrosinase-inhibiting effects comparable to the positive control kojic acid.

## 2. Results and Discussion

### 2.1. Strain Identification

The fungal strain SCSIO 40433 was isolated from the glacier sediment sample collected in the Arctic region (E 14.211086°, N 77.955638°). The ITS region of the rDNA of strain SCSIO 40433 (GenBank Accession number MT656013) was amplified, and sequenced, allowing the construction of a phylogenetic tree by the neighbor joining method (Supplementary Materials Figure S1). Analysis of the phylogenetic tree clearly showed that SCSIO 40433 should be a species of *Tolypocladium cylindrosporum*, given the perfect match with other identical species in GenBank (MH399740, MH399741, NR-167967, MG228379 and MG228380) [28,29].

### 2.2. Compound Isolation and Structure Elucidation

After culture-production optimization, the ISP4 liquid medium was selected for fermentation of *T. cylindrosporum* SCSIO 40433. Crude extracts were obtained from 12.8 L fermentation broths of *T. cylindrosporum* SCSIO 40433 cultured for 14 days in ISP4 liquid media. Multiple-step chromatographic isolation of the crude extracts afforded cylindromicin (**1**) and seven known compounds (**2**–**8**, Figure 1).

**Figure 2:**
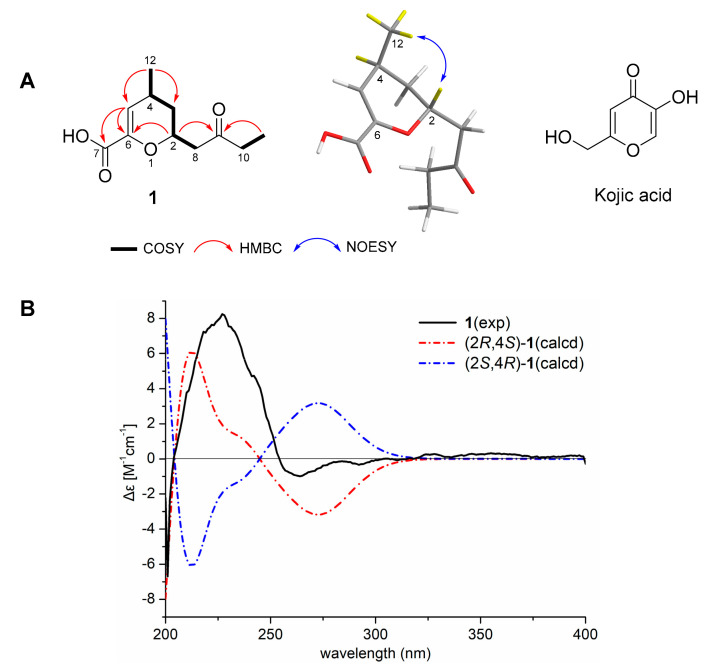
Selected key COSY, HMBC, and NOESY correlations of compound **1** (**A**) and comparison of experimental ECD spectra of **1** with the calculated one for 2*R*, 4*S* configurations (**B**).

Cylindromicin (**1**) was isolated as a light brown oil. The molecular formula of **1** was determined as C_11_H_16_O_4_, according to the ion peak at *m*/*z* 213.1116 [M + H]^+^ (calcd. for C_11_H_17_O_4_ 213.1121, Figure S2) by high-resolution electrospray ionization mass spectrometry (HRESIMS), indicating four degrees of unsaturation. The UV spectrum of **1** displayed a maximum absorbance wavelength at 236 nm (Figure S3). The IR spectrum suggested the presence of hydroxy (3412 cm^−1^), carbonyl (1705 cm^−1^), alkene (1643 cm^−1^), and ether (1224 cm^−1^) functional groups (Figure S4). The ^1^H and ^13^C NMR data of **1** (Table 1, Figures S5–S8) were similar to those of the synthetic compound trans-2-butyl-6-methoxycarbonyl-4-methyl-3,4-dihydro-2H-pyran (**9**) (Figure 1) [30]. Comparison of spectroscopic data of **1** and **9** showed that compound **1** was different from **9** due to the presence of a carbonyl group (δ_C_ 211.60) and the absence of a methoxyl group (δ_C_ 51.9) (Figure S6). Different from the *n*-butyl group at C-2 in **9**, a butan-2-one alkyl side chain was located at C-2 in **1**. This assignmenet was confirmed by the ^1^H-^1^H correlation spectroscopy (COSY) correlations between H-10/H-11 and H-8/H-2/H-3/H-4/H-12, and heteronuclear multiple bond correlation (HMBC) correlations between H-2 and C-6/C-9 and between H-11 and C-9 (Figure 2A, Figures S10 and S11). The location of a carboxyl group at C-6 in **1**, different from the methoxycarbonyl group at C-6 in **9**, was supported by the HMBC correlations between H-5 and C-6/C7 (Figure 2A and Figure S10). The trans-location of H-2 and H-4 on the 6-membered 3,4-dihydro-2H-pyran ring in **1** was deduced from the key NOESY correlations between H-12 and H-2 (Figure 2A and Figure S11). Subsequently, the ECD calculations were perfomed on (2*R*,4*S*)-**1** and (2*S*,4*R*)-**1**, respectively. It was found that the experimental ECD spectrum of **1** was consistent with that calculated for (2*R*,4*S*)-**1** (Figure 2B and Figure S13, Tables S1–S3). Therefore, the absolute configurations of **1** were assigned as 2*R*,4*S*.

Compounds **2**–**8** were identified as tolypoalbin (**2**) [31], epi-citrinin H1 (**3**) [32], dicitrinin A (**4**) [33], penicitrinone A (**5**) [34], citreorosein (**6**) [35], phenol A (**7**) [36] and phenol A acid (**8**) [37] by comparing their NMR and MS data with those reported in the literature.

### 2.3. Bioactivity Evaluation of Compounds **1**–**8**

All the isolated compounds in this study were evaluated for the tyrosinase inhibition activity. Cylindromicin (**1**) exhibited significant tyrosinase inhibition activities at the concentrations of 20 and 40 μM (Table 2), which were comparable to those of the positive control kojic acid. None of the compounds **2**–**8** showed tyrosinase inhibition activities (Table S4). Compounds **1**–**8** were also evaluated for antibacterial and alpha-glucosidase inhibition activity. Unfortunately, no such activities were observed.

### 2.4. Structure-Activity Comparsion of Cylindromicin (**1**) and Kojic Acid

The core unsaturated pyran ring of **1** was structurally similar to kojic acid (Figure 2A) [22], which was probably the reason for the tyrosinase inhibition activity of **1**. Structure differences between **1** and kojic acid were the functionalities substituted on the core ring. Kojic acid was reported to form strong hydrogen bonds with the residues at the entrance of the active site of tyrosinase [21]. Similarly, the carboxyl group at C-6 and carbonyl group at C-9 of **1** presumably played important roles in the tyrosinase inhibition activity. Other fungi-derived tyrosinase inhibitors, such as 6-*n*-pentyl-α-pyrone [38], and two benzofuran derivatives [39], were found to possess unsaturated pyran or furan ring.

## 3. Materials and Methods

### 3.1. General Experimental Procedures

Optical rotations were obtained using a 341 Polarimeter (Perkin-Elmer, Inc. Norwalk, CT, USA). The CD spectra were obtained on a Chirascan circular dichroism spectrometer (Applied Photophysics, Ltd. Surrey, UK). UV spectra were recorded on a UV-2600 spectrophotometer (Shimadzu, Tokyo, Japan). IR spectra were recorded on an Affinity-1 FT-IR spectrometer (Shimadzu, Tokyo, Japan). The 1D and 2D NMR spectra were collected using a Bruker AV-700 MHz NMR spectrometer (Bruker Biospin GmbH, Rheinstetten, Germany) at 700MHz for ^1^H NMR and 175 MHz for ^13^C NMR with tetramethylsilane (TMS) as the internal standard. High-resolution electrospray ionization mass spectra (HRESIMS) were measured on a quadrupole-time-of-flight mass spectrometer (Bruker Maxis 4G). Column chromatography were performed with silica gel (100–200 mesh, 300–400 mesh; Jiangyou Silica gel development, Inc. Yantai, China) or Sephadex LH-20 (40–70 μm, Amersham Pharmacia Biotech AB, Uppsala, Sweden). Medium pressure liquid chromatography (MPLC) was carried out using a CEETAH flash system (Bonna-Agela technologies Inc. Tianjin, China). Compounds were detected with a 1260 Infinity HPLC (Agilent Inc. USA). Semi-preparative HPLC were performed on reverse phase columns (XB-C18, 250 × 10 mm, 5 µm, Agilent Inc. Palo Alto, CA, USA; or ACE Excel Super C18, 250 × 10 mm, 5 µm, Advanced Chromatography Technologies Co. LTD, Aberdeen, UK) using a Hitachi HPLC station (Hitachi-L2130, Tokyo, Japan) equipped with a diode array detector (DAD, Hitachi L-2455).

### 3.2. Isolation and Identification of Strain SCSIO 40433

The glacier sediment sample was air-dried aseptically in a laminar flow clean bench. Secondly, two grams of the air-dried sample was suspended in eighteen milliliters of sterile seawater. The sample solution was serially diluted (tenfold) with sterile water and spread on plates containing ISP4 agar medium (Starch 1%, (NH_4_)_2_SO_4_ 0.2%, K_2_HPO_4_ 0.1%, CaCO_3_ 0.1%, MgSO_4_·7H_2_O 0.1%, trace elements (FeSO_4_·H_2_O 0.1%, MnCl_2_·4H_2_O 0.1%, ZnSO_4_·7H2O 0.1%) 0.1%, Agar 1.5%) and PDA (Potato Dextrose Agar) medium. Isolation plates were incubated for 2 weeks at 28 °C. After two weeks of incubation, some microbes were observed as circular white colonies on both kinds of plates. The pure fungal spores were streaked on fresh ISP4 agar and PDA plates and incubated for another 2 weeks at 28 °C. It was clearly observed that the growth of isolated microbes on PDA plates was slower than those on ISP4 plates. The pure isolated strain was obtained by repeated inoculation of monoclonal colonies onto fresh modified ISP4 agar plate. It was identified as *T. cylindrosporum* on the basis of the phylogenetic tree. Isolation of genomic DNA, amplification of the ITS region by polymerase chain reaction (PCR), sequence comparison, and phylogenetic tree construction of the strain SCSIO 40433 were performed as described previously [40]. The strain SCSIO 40433 could grow slowly at 4 °C on ISP4 plates. Salinity dependence on the growth of SCSIO 40433 was not observed when 3% sea salt was supplemented in the media.

### 3.3. Fermentation and Extraction of Strain SCSIO 40433

Initially, four different kinds of media were used to optimize the fermentation conditions of *T. cylindrosporum* SCSIO 40433, including potato dextrose broth (PDB), ISP4 culture medium (Starch 1%, (NH_4_)_2_SO_4_ 0.2%, K_2_HPO_4_ 0.1%, CaCO_3_ 0.1%, MgSO_4_·7H_2_O 0.1%, trace elements (FeSO_4_·H_2_O 0.1%, MnCl_2_·4H_2_O 0.1%, ZnSO_4_·7H_2_O 0.1%), Petter broth medium (glucose 2%, yeast extract 0.3%, malt Extract 0.3%, peptone 0.5% and sea salt 3%) and solid-state rice medium. After 14 days of incubation at 28 °C, a higher production of biomass and secondary metabolites, determined by HPLC analysis, was clearly observed on the ISP4 culture medium.

*Tolypocladium cylindrosporum* SCSIO 40433 was cultured on ISP4 agar plates at 28 °C for 8 days. Subsequently, two or three pieces of mycelial agar plugs were inoculated in 250 mL Erlenmeyer flasks, each containing 50 mL ISP4 culture medium. After 8 days of incubation at 28 °C on a rotary shaker at 180 rpm, 2 mL seed cultures were transferred into sixty-four 1000 mL Erlenmeyer flasks, each containing 200 mL ISP4 culture medium. The scaled-up fermentation of SCSIO 40433 was kept on for 14 days at 28 °C and 180 rpm, on a rotary shaker.

The 12.8 L fermentation broth of SCSIO 40433 were separated to supernatant and mycelia by centrifugation at 4000 rpm for 10 min. The supernatant was thoroughly extracted with an equal volume of ethyl acetate (EtOAc) three times. Mycelia were extracted by 70% acetone/water (*v*/*v*) mixture solvent to afford mycelia extract. After removing the acetone from mycelia extract, the residue solvent was extracted with equal volume of EtOAc. The crude extracts obtained from the supernatant and mycelia were almost identical, according to the HPLC analysis. Therefore, the EtOAc extracts of the supernatant and mycelia were combined and concentrated by rotary evaporator under vacuum at a temperature not exceeding 32 °C to give crude extract (7.0 g).

### 3.4. Purification of the Compounds ***1***–***8***

The crude extract was subjected to a reverse phase C18 column (YMC*GEL ODS-A, 120A S-5 μm, 310 × 45 mm) using MPLC with gradient elution for 120 min (acetonitrile/water system, from 0 to 100% acetonitrile). MPLC chromatography yielded 10 fractions which were collected and named as F1–F10. Chemical constituents of each fraction were analyzed by TLC under ultraviolet (λ = 254 nm) and HPLC equipped with DAD detector, respectively. Fractions F5–F10 were subjected to Sephadex LH-20 column and eluted with the solvent (chloroform: methanol = 1:1, *v*/*v*) to afford sub-fractions. Fraction F5 (155 mg) afforded eleven sub-fractions by Sephadex LH-20 chromatography. Subsub-fractions F5-4 to F5-7 were combined under the guidance of TLC analysis and produced 25 mg residue after evaporation of solvent. The abovementioned residue of F5-4~7 was purified by semi-preparative HPLC (ACE Excel 5 Super C18, 250 × 10 mm; isocratic elution (25 % acetonitrile-water) to give compound **1** (2.8 mg, *t*_R_ = 7.1 min) and compound **2** (5.5 mg, *t*_R_ = 14.23 min) with a flow rate of 2.5 mL/min and UV detection at 230 and 274 nm. Fraction F6 (92 mg) was separated by Sephadex LH-20 column again. Subsub-fractions F6-6 to F6-10 were combined and purified by semi-preparative HPLC to yield compounds **3** (2.27 mg, *t*_R_ = 12.20 min) and **4** (4.8 mg, *t*_R_ = 13.10 min) with a flow rate of 2.5 mL/min, and UV detection at 244 and 400 nm. Fractions F7 and F8 (172 mg) were combined and loaded onto Sephadex LH-20 column to provide sub fractions. F7-F8-5 to F7-F8-11 were combined under the guidance of TLC analysis and further purified by semi-preparative HPLC to afford compound **5** (3.3 mg, *t*_R_ = 14.33 min) and compound **6** (3.5 mg, *t*_R_ = 16.42 min) with a flow rate of 2.5 mL/min, and UV detection at 244 and 374 nm. Similarly, compound **7** (12 mg, *t*_R_ = 8.26 min) and **8** (2.5 mg, *t*_R_ = 10.50 min) were separated by semi-preparative HPLC from fraction F9 with the same flow rate, and UV detection at 230 and 274 nm.

Cylindromicin (**1**): light brown oil; [*α*]${}_{D}^{25}$ +39.28 (*c* 0.14, MeOH); IR (film) *ν*_max_: 3412, 2927, 1705, 1643, 1456, 1224, 773 cm^−1^, (see Supplementary Figure S3); UV (MeOH) λ_max_ (*log ε*): 236 (3.78) nm; CD (0.25 mg/mL, MeOH) (Δ*ε*) 227 (8.25) nm; ^1^H and ^13^C NMR data, see Table 1; (+)-HR-ESI-MS *m*/*z* 213.1116 [M + H]^+^ (calcd. for C_11_H_16_O_4_ [M + H]^+^, 213.1121)

### 3.5. Computational Method for ECD Prediction

Time-dependent density functional theory electronic circular dichroism (TDDFT-ECD) calculation has been proven to be an efficient method for the stereochemistry study of the molecules which have achiral chromophores in a chiral environment [41]. The conformational analysis of (2*R*,4*S*)-**1** were performed by Sybyl 8.1 software using MMFF94s force field, which afforded the conformers for (2*R*,4*S*)-**1** with an energy cutoff of 3.0 kcal mol^-1^ to the global minima. All of the obtained conformers were optimized by Gaussian09 software at the B3LYP/6-31+G(d) level in the gas phase. TDDFT-ECD calculations for the optimized conformers were performed at the B3LYP/6-31+G(d) level in methanol using the polarizable conductor calculation model (PCCM) [42]. The overall ECD curves of all the conformers were weighted by Boltzmann distribution after a UV correction of 0 nm. The ECD curves were produced by SpecDis 1.6 software with sigma = 0.3 eV [43].

### 3.6. Tyrosinase Inhibition Assay of Compounds ***1***–***8***

Tyrosinase inhibition activities of compounds **1**–**8** were determined using kojic acid as the positive control and DMSO as the negative control. Tyrosinase inhibition assays were carried out according to previously reported method [44]. Firstly, 20 mmol/L stock solutions of all isolated compounds were prepared and then diluted to different concentrations using dimethyl sulfoxide (DMSO) as the solvent. Tyrosinase (Sigma-Aldrich, catalog number T3824, Saint Louis, USA) and L-DOPA were dissolved in 50 mmol/L phosphate buffer (pH 6.8). A total of 150 μL of substrate solution (1 mmol/L) and 10 μL of sample solution were added to 96 well microtiter plates. Subsequently, 40 μL of tyrosinase solution (100 U/mL) was quickly added to each well and gently mixed. After thorough mixing by vortex, initial absorbance of the assay solution was measured immediately at 475 nm. The second absorbance of the assay solution was measured after 20 min incubation at 30 °C. The inhibition activity of enzyme was recorded by calculation of the optical density (OD475) differences at a wavelength of 475 nm. The relative inhibition rate and molar extinction coefficient of the product at 475 nm was calculated as 3700 mol L^−1^ cm^−1^.

Relative inhibition rate (%) = (1−ΔAm /ΔAc) × 100%

where ΔAm indicates the enzyme activity of the sample and ΔAc is the enzyme activity of the control (sample was replaced by an equal volume of solvent). The experiments were performed in triplicate followed the same protocol and procedure to make sure of the inhibitory activity of isolated compounds.

### 3.7. Antibacterial and Alpha-Glucosidase Inhibition Assay of Compounds ***1***–***8***

Compounds **1**–**8** were evaluated for antibacterial activities by the paper-disc diffusion method [45]. The indicator bacteria strains included two Gram-negative bacteria *Acinetobacter baumannii* ATCC 19606 (American type culture collection, Manassas, VA, USA), *Escherichia coli* ATCC 25922 (American type culture collection, Manassas, VA, USA) and three Gram-positive bacteria *Staphylococcus aureus* ATCC 29213 (American type culture collection, Manassas, VA, USA), methicillin-resistant *S. aureus* (MRSA) shhs-A1 (South China Sea Institute of Oceanology, Guangzhou, China), and *Micrococcus luteus* SCSIO ML01 (South China Sea Institute of Oceanology, Guangzhou, China). The concentrations of compounds **1**–**8** were prepared as 2.56 mg/mL. Vancomycin and trimethoprim were used as positive controls, while DMSO was used as a negative control in the antibacterial assay. Furthermore, compounds **1**‒**8** were evaluated for alpha-glucosidase inhibition activity [46]. The concentrations of the samples were prepared as 20 μM. Acarbose was used as a positive control while DMSO was used as a negative control in the enzymatic assay.

## 4. Conclusions

Polar region-derived microorganisms are emerging as attractive resources to produce new natural products and unique enzymes. However, most of the polar region-derived microbial natural products were characterized from the Antarctic-derived microorganisms to date. Our data showed that *T. cylindrosporum* SCSIO 40433, derived from Arctic region, could produce a new compound, cylindromicin (**1**), a bioactive inhibitor against tyrosinase. Tyrosinase is a critical rate-limiting enzyme in the biosynthesis of melanin. It is widely found in microorganisms, animals, plants, and human bodies. Kojic acid is a well-known tyrosinase inhibitor widely used as a skin lighting/depigmenting agent in the cosmetic industries [21]. Interestingly, cylindromicin (**1**) exhibited significant tyrosinase inhibition activities that were comparable to those of kojic acid. Unfortunately, compounds **1**–**8** showed no antibacterial and alpha-glucosidase inhibition activities. In conclusion, these data indicated that Arctic-derived microorganisms should have potential in searching for novel and bioactive secondary metabolites.

## Figures and Tables

**Table 1:** ^1^H (700 MHz) and ^13^C NMR data (175 MHz) of cynlindromicin (**1**, in CD_3_OD).

Carbon No.	*δ*_C_, Type	*δ*_H_ (Multi, *J* in Hz)
2	70.6, CH	4.38 (m)
3	37.5, CH_2_	1.60 (m), 1.70 (m)
4	26.2, CH	2.41 (m)
5	117.1, CH	6.02 (d, 4.2)
6	144.6, C	
7	166.9, C	
8	47.5, CH_2_	2.64 (dd, 5.6,16.8); 2.93 (dd, 7.0,16.8)
9	211.6, C	
10	34.8, CH_2_	2.56 (m)
11	7.8, CH_3_	1.03 (t, 7.0)
12	22.0, CH_3_	1.11 (d, 7.0)

**Table 2:** Tyrosinase inhibition rate (%) of compound **1** at different concentrations (μM).

Concentrations	1	Kojic Acid
10 μM	0.62 ± 0.04%	1.23 ± 0.06%
20 μM	7.41 ± 0.06%	6.17 ± 0.09%
40 μM	23.4 ± 0.11%	27.41 ± 0.09%

## Data Availability

All data and figures generated or used during the study appear in the submitted article.

## Supplementary Materials

The original spectra (Figures S1–S13), ECD calculation results (Tables S1–S3) of **1,** and tyrosinase inhibition assay (Table S4) of compounds **2**–**8** are available online.

## Sample Availability

Samples of the compounds **1**–**8** are available from the authors.
