# Supplementary material for: Cylindromicin from Arctic-Derived Fungus Tolypocladium sp. SCSIO 40433
Source: Molecules. 2021 Feb 18;26(4):1080. doi: 10.3390/molecules26041080 (PMC7922244; doi:10.3390/molecules26041080)
Supplement: Supplementary file 1 [file molecules-26-01080-s001.pdf]

Supplementary Material

Cylindromycin from Arctic-derived fungus *Tolypocladium* sp. SCSIO 40433

Imran Khan [1,2], Jing Peng [1,2], Zhuangjie Fang [1,2], Wei Liu [1,3], Wenjun Zhang [1,2,3,4], Qingbo Zhang [1,3,4], Liang Ma [1,3], Guangtao Zhang [1,3,4], Changsheng Zhang [1,2,3,4,*] and Haibo Zhang [1,3,4,*]

[1] Key Laboratory of Tropical Marine Bio-resources and Ecology, Guangdong Key Laboratory of Marine Materia Medica, RNAM Center for Marine Microbiology, South China Sea Institute of Oceanology, Chinese Academy of Sciences, 164 West Xingang Road, Guangzhou 510301, China.; imranmb_kust@yahoo.com; urnotpengjing@163.com; 991653913@qq.com; 448242066@qq.com; maliangyc@hotmail.com

[2] University of Chinese Academy of Sciences, Beijing 100049, China; wzhang@scsio.ac.cn

[3] Southern Marine Science and Engineering Guangdong Laboratory (Guangzhou), 1119 Haibin Road., Nansha District, Guangzhou 511458, China; gudaobo@163.com; gtzhang@scsio.ac.cn

[4] Sanya Institute of Oceanology, SCSIO;

* Correspondence: czhang@scsio.ac.cn; zhanghb@scsio.ac.cn; Tel.: +86-020-89108532

Table of Contents

**Contents**	**Pages**
**Figure S1**. Phylogenetic tree of *Tolypocladium cylindrosporum* SCSIO 40433 based on neighbour-joining method	S4
**Figure S2**. The HRESI-MS of cylindromicin (**1**)	S5
**Figure S3**. The UV spectrum of cylindromicin (**1**)	S5
**Figure S4**. The CD spectrum of cylindromicin (**1**)	S6
**Figure S5**. The IR spectrum of cylindromicin (**1**)	S6
**Figure S6**. The ^{1}H NMR spectrum of cylindromicin (**1**)	S7
**Figure S7**. The ^{13}C-NMR spectrum of cylindromicin (**1**)	S8
**Figure S8**. The DEPT-135 spectrum of cylindromicin (**1**)	S9
**Figure S9**. The HSQC spectrum of cylindromicin (**1**)	S10

**Figure S10**. The ^{1}H-^{1}H COSY spectrum of cylindromicin (**1**)	S11
**Figure S11**. The HMBC spectrum of cylindromicin (**1**)	S12
**Figure S12**. The NOESY spectrum of cylindromicin (**1**)	S13
**Figure S13**. Most stable conformers of (2*R*, 4*S*)-**1**.	S14
**Table S1**. Gibbs free energiesa and equilibrium populationsb of low-energy conformers of (2*R*, 4*S*)-**1**.	S15
**Table S2**. Energies of (2*R*, 4*S*)-**1** at B3LYP/6-31+G(d) in gas phase.	S15
**Table S3**. Cartesian coordinates for the low-energy reoptimized MMFF conformers of (2*R*, 4*S*)-**1** at B3LYP/6-311+G(d) level of theory in CH_3OH.	S15
**Table S4**. Tyrosinase inhibition rate (%) of compounds **2–8** at different concentrations (μM).	S19

Figure S1. Phylogenetic tree of *Tolypocladium cylindrosporum* SCSIO 40433 based on neighbour-joining method

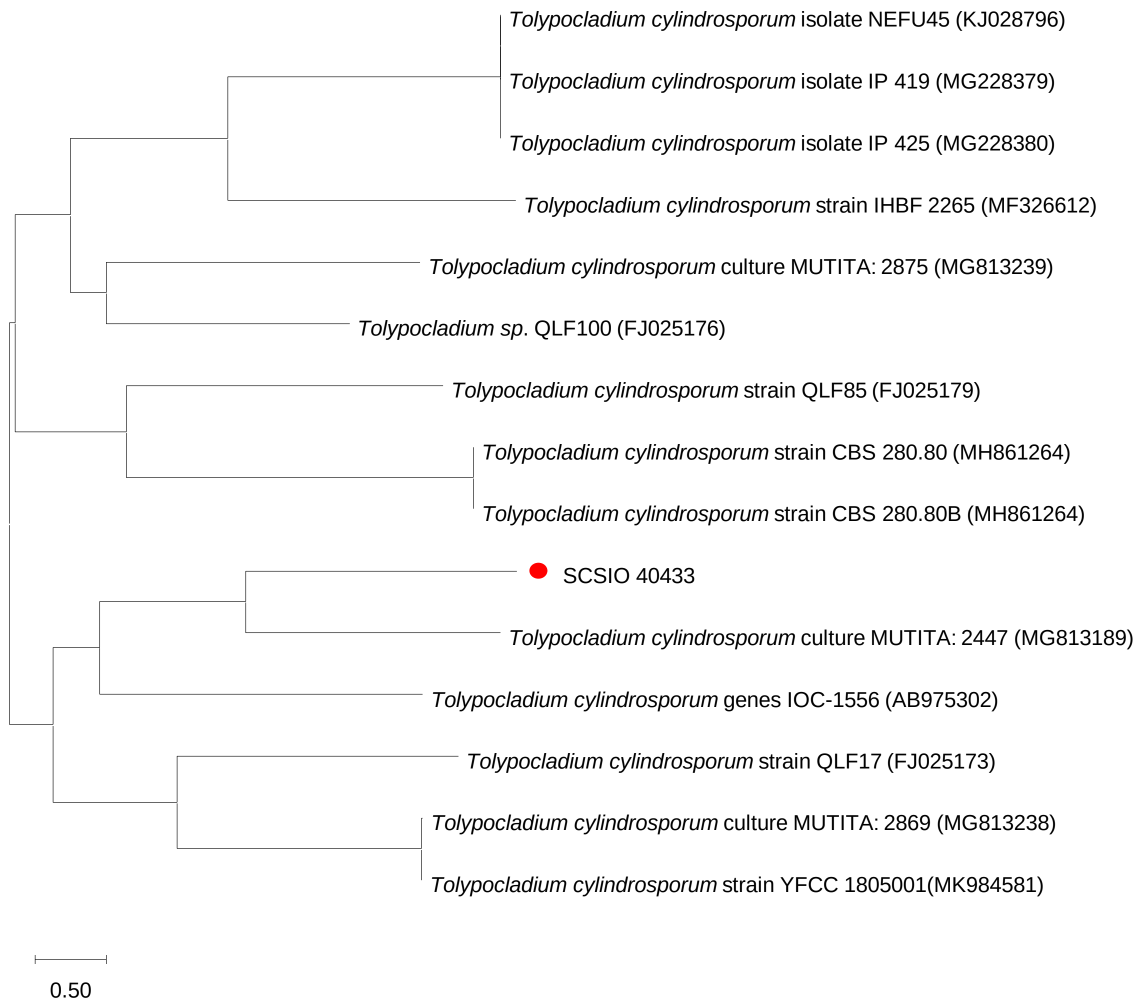

ITS sequence of *Tolypocladium cylindrosporum* SCSIO 40433

ATACCCAACGTTGCTTCGGCGGGACCGCCCCGGCGCCCTCGGCGTCCCGGAACCAGGCG
CCCGCCGGAGGACCCAAACTCTTGTTTAAACCATAGTGGCATATTCTGAGTCTCACAAGA
AAAATGAATCAAAACTTTCAACAACGGATCTCTTGGCTCTGGCATCGATGAAGAACGCA
GCGAAATGCGATAAGTAATGTGAATTGCAGAATTCAGTGAATCATCGAATCTTTGAACG
CACATTGCGCCCGCCAGTATTCTGGCGGGCATGCCTGTTCGAGCGTCATTTCAACCCTCA
AGCCCCAGCCGGCTTGGTGTTGGGGACCGGCCCCGGCCGCCCCCCAAATGCAGTGGCGA
CCTCGCCGCAGCCTCCCCTGCGTAGTAGCACAACCTCGCACCGGAGCGCGGAGACGGTC
ACGCCGTAAAACGCCCAACTTCTCAGAGTTGACCTCGGATCAGGTAGGAATACCCGCTG
AA

Figure S2. The HRESI-MS of cylindromicin (**1**)

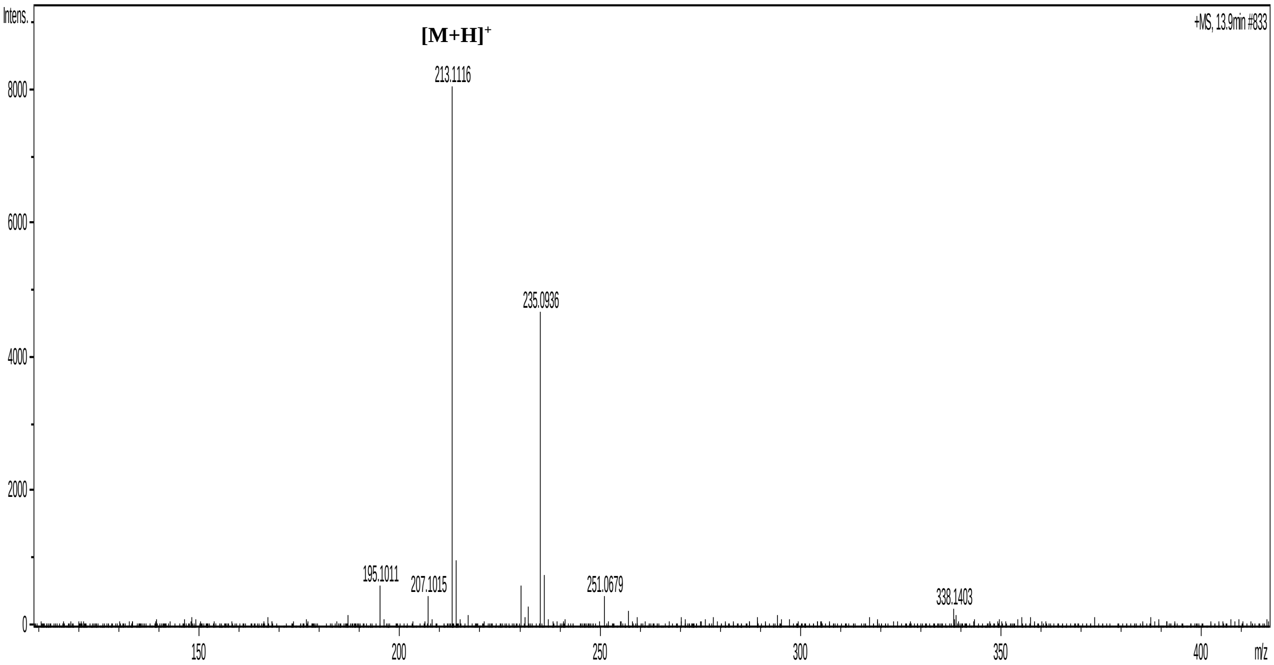

Figure S3. The UV spectrum of cylindromicin (**1**)

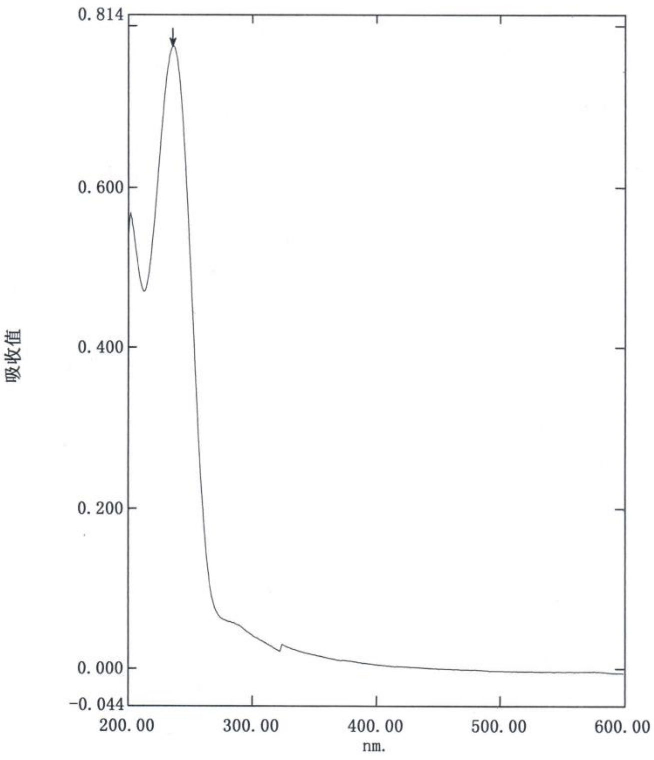

[测定属性]
波长范围 (nm.): 200. 00 到 600. 00
扫描速度: 中速
采样间隔: 0. 5
自动采样间隔: 启用
扫描模式: 单个

[仪器属性]
仪器类型: UV-2600 系列
测定方式: 吸收值
狭缝宽: 2. 0
积分时间: 0. 1 秒.
光源转换波长: 323. 0 nm
检测器单元: 直接
S/R 转换: 标准
阶梯校正: OFF

[附件属性]
附件: 无

[数据处理参数]
阈值: 0. 0100000
点: 5
内插: 停用
平均: 停用

[样品准备属性]
重量:
体积:
稀释:
光程长:
附加信息:

No.	P/V	波长(nm)	吸收值	描述
1	⬆	236. 50	0. 775	

Figure S4. The CD spectrum of cylindromicin (**1**)

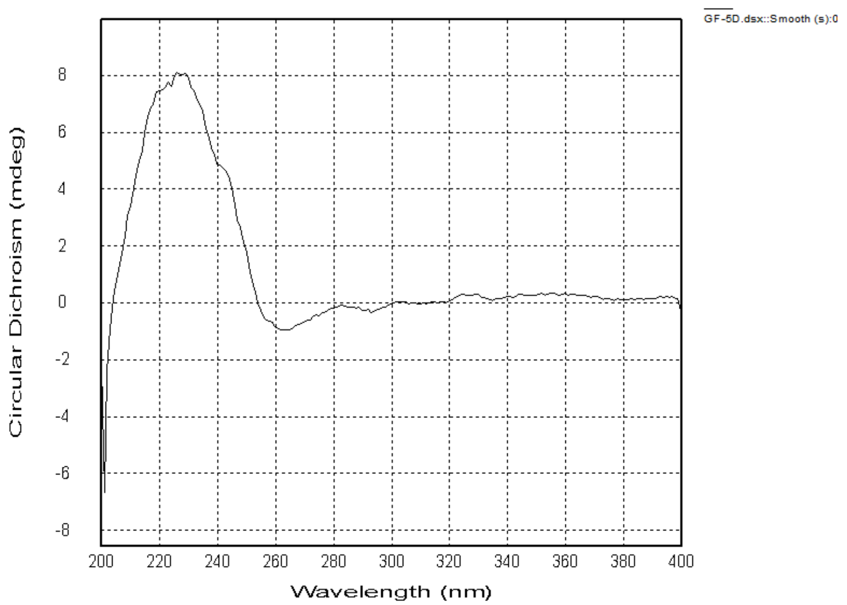

Figure S5. The IR spectrum of cylindromicin (**1**)

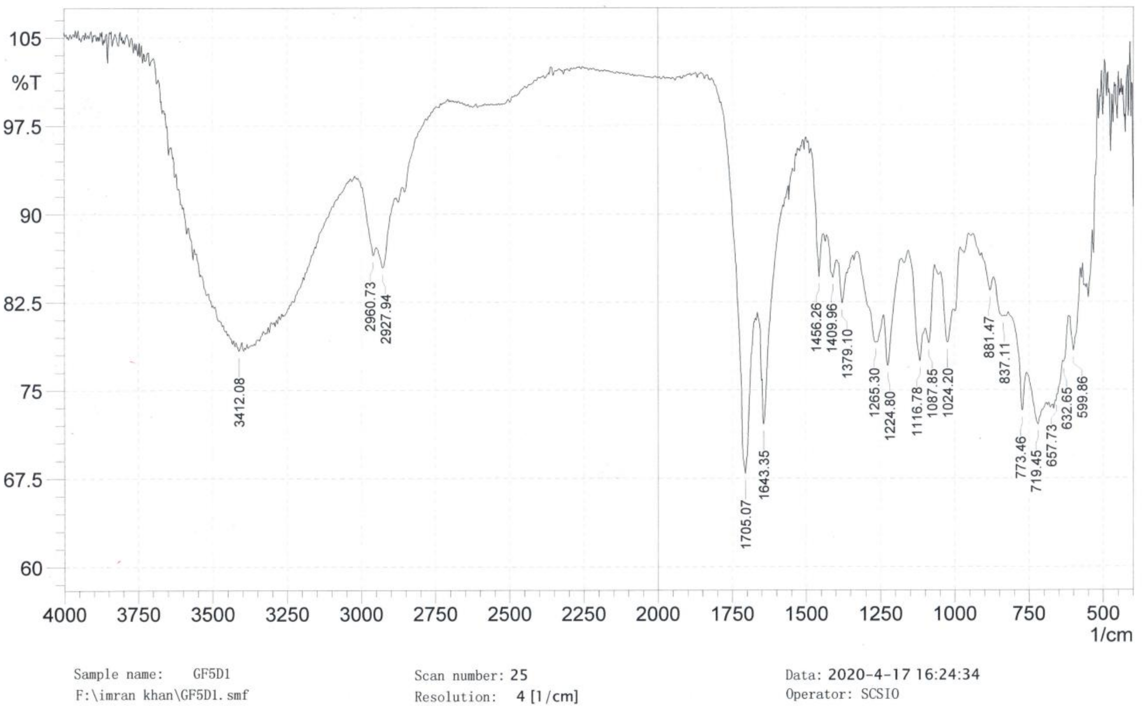

Figure S6. The ^{1}H NMR spectrum of cylindromicin (**1**)

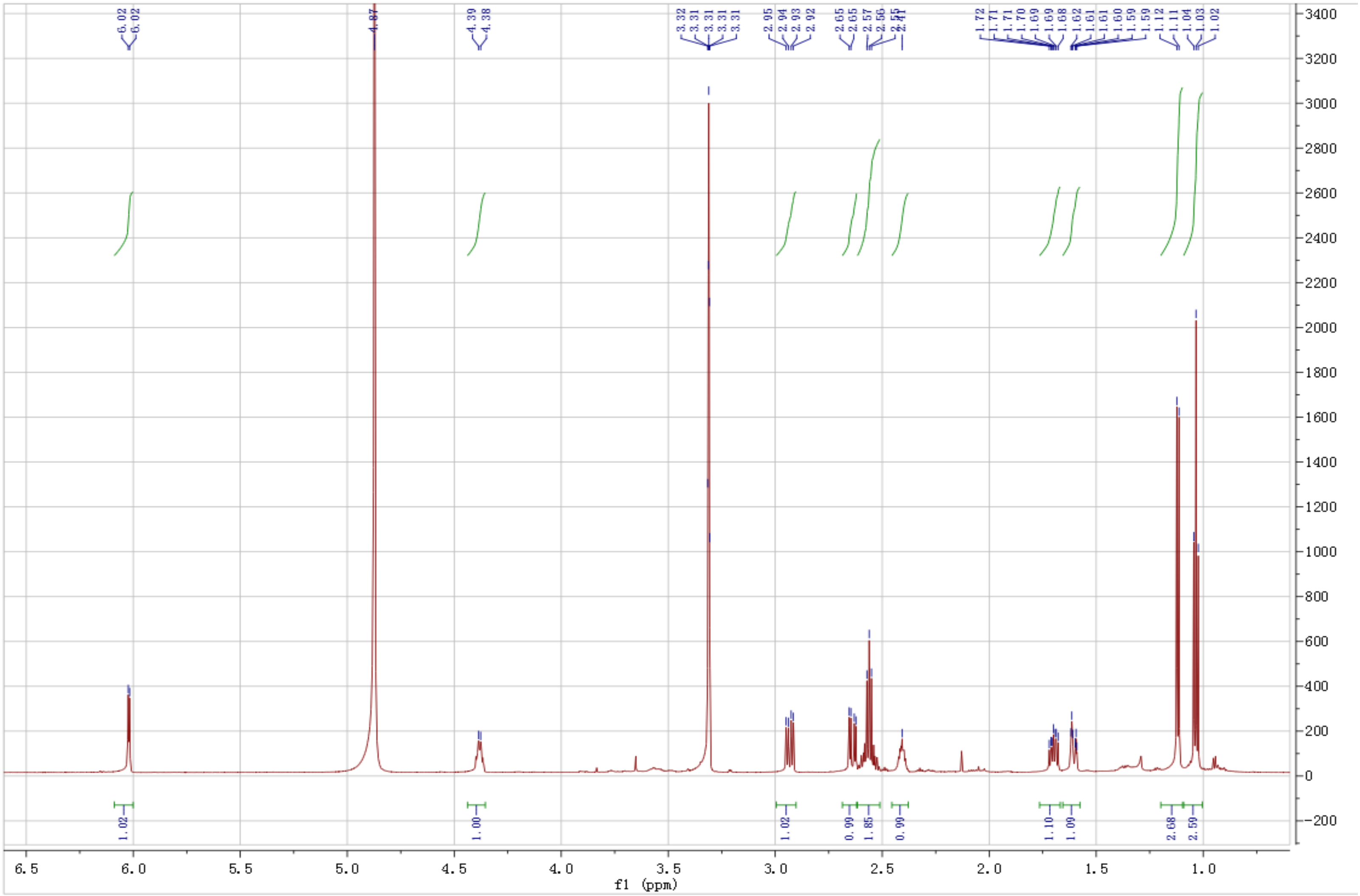

Figure S7. The ^{13}C-NMR spectrum of cylindromicin (**1**)

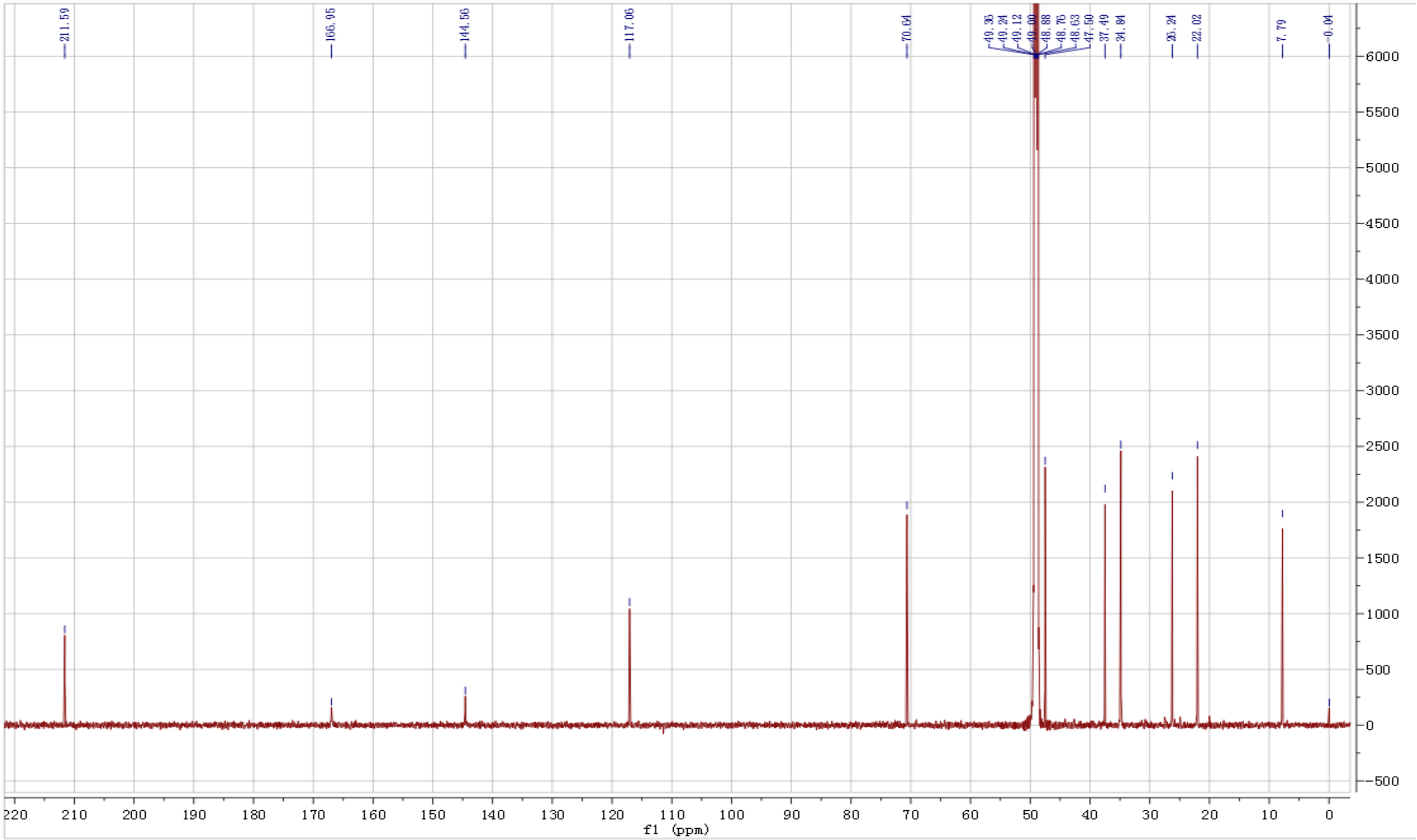

Figure S8. The DEPT-135 spectrum of cylindromicin (**1**)

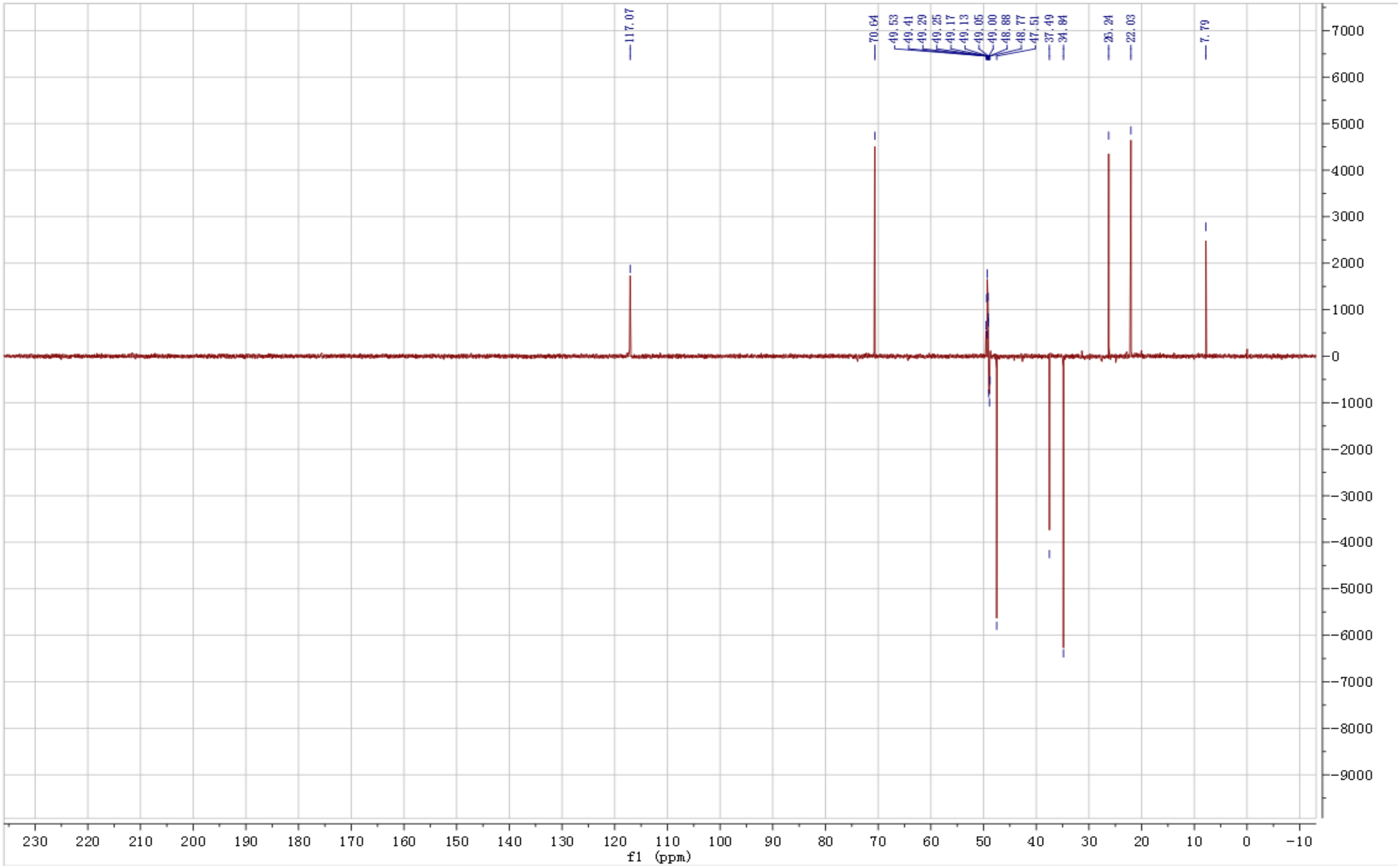

Figure S9. The HSQC spectrum of cylindromicin (**1**)

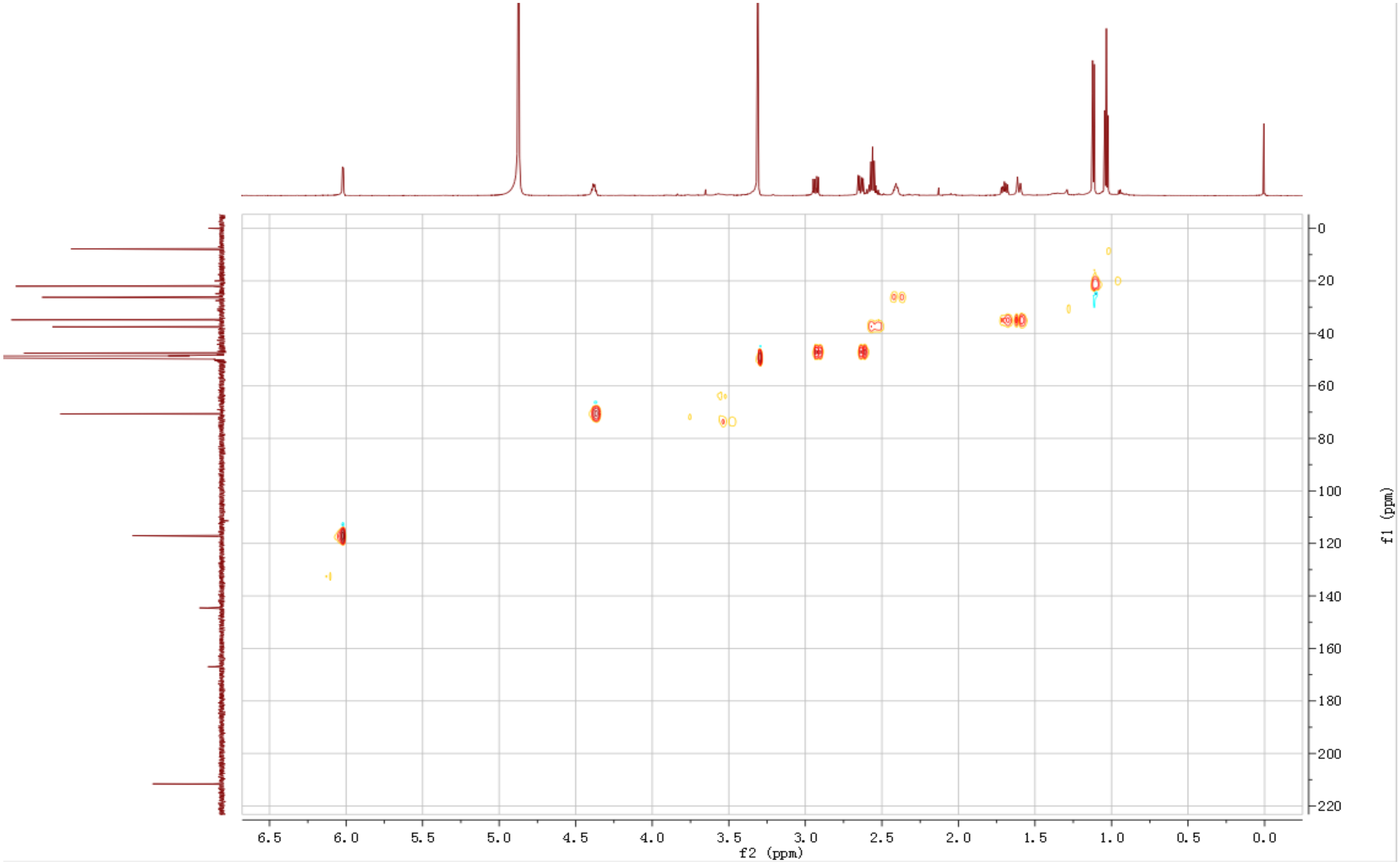

Figure S10. The ^{1}H-^{1}H COSY spectrum of cylindromicin (**1**)

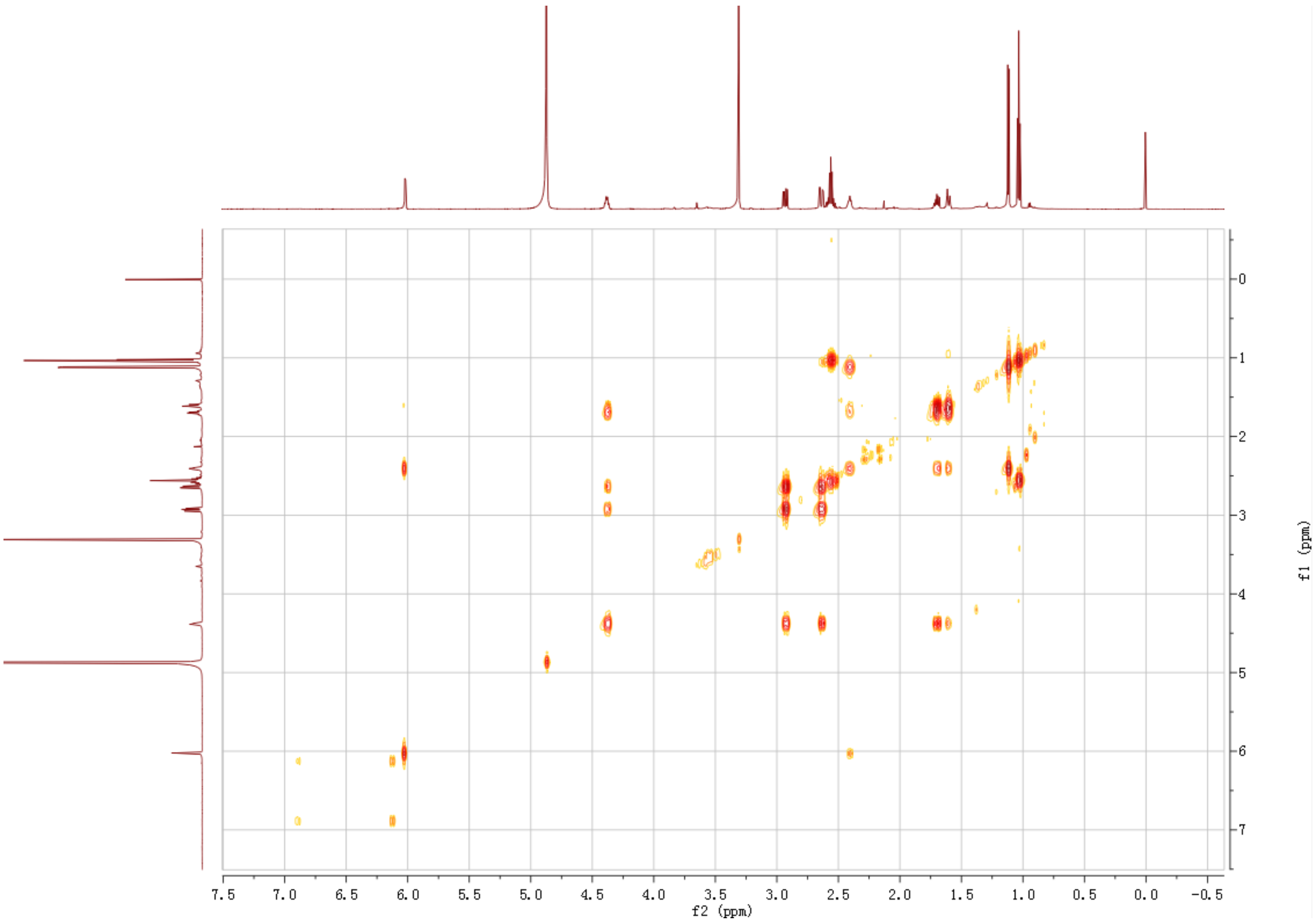

Figure S11. The HMBC spectrum of cylindromicin (**1**)

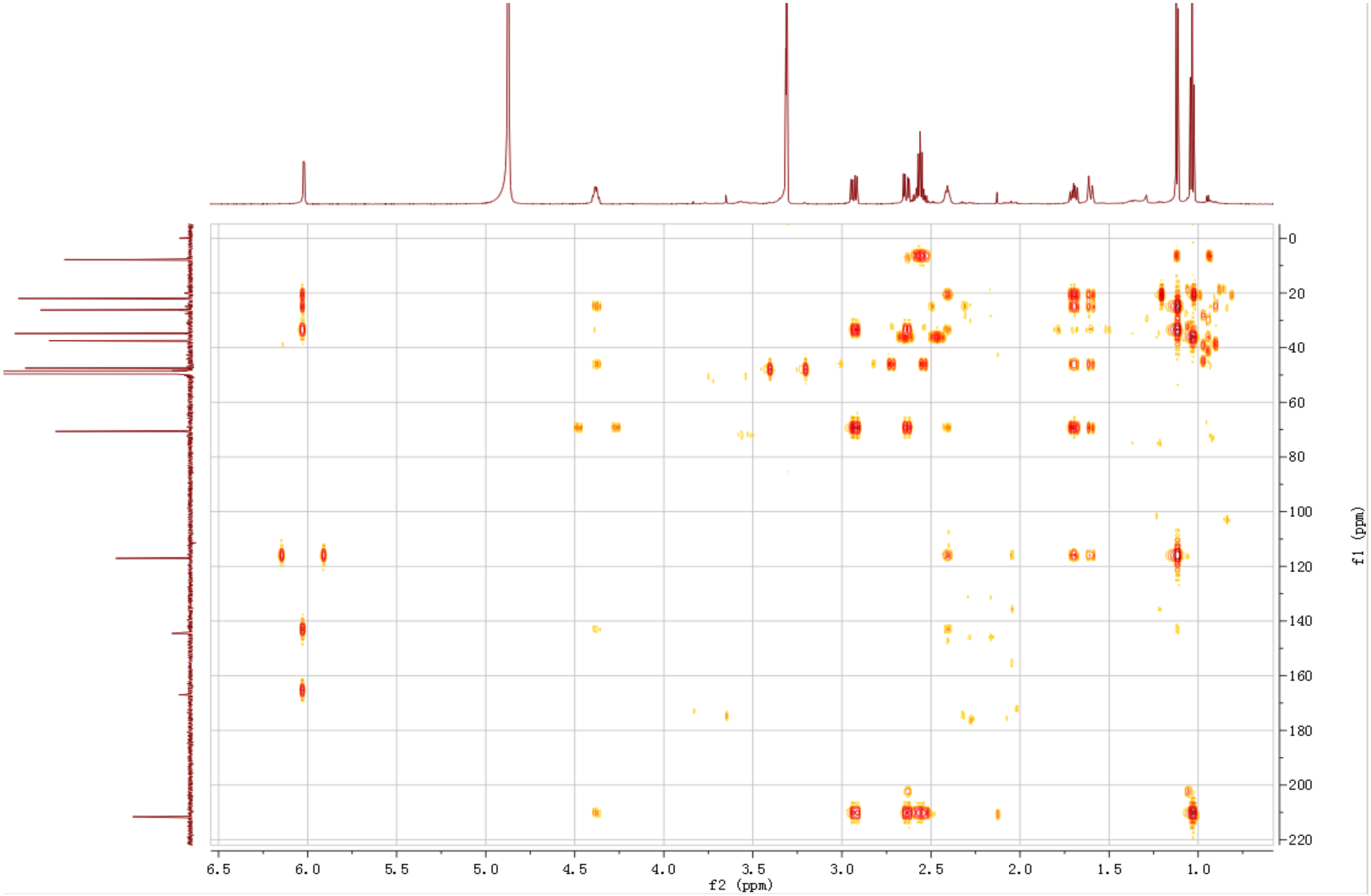

Figure S12. The NOESY spectrum of cylindromicin (**1**)

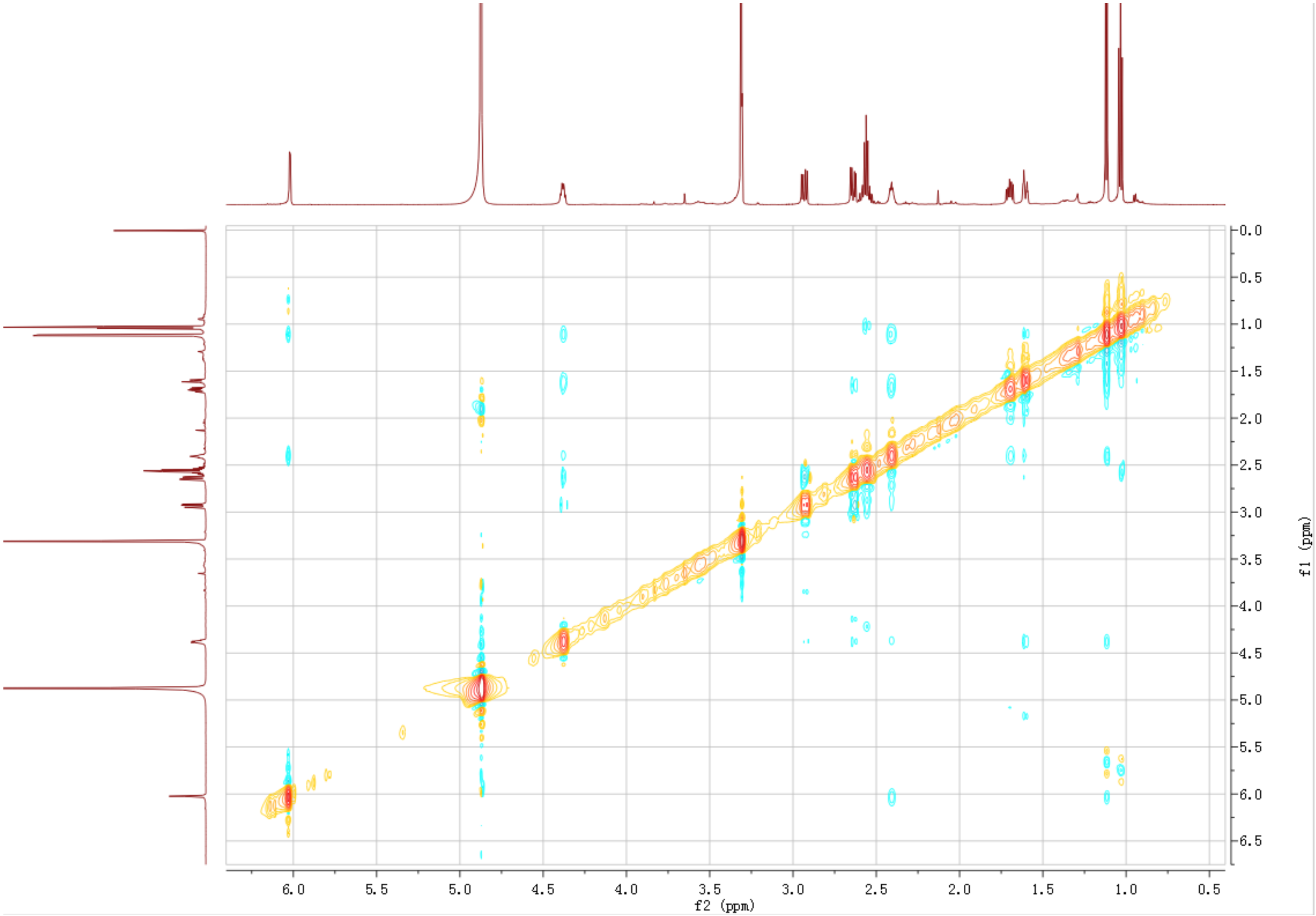

Figure S13. Most stable conformers of (2*R*, 4*S*)-**1**.

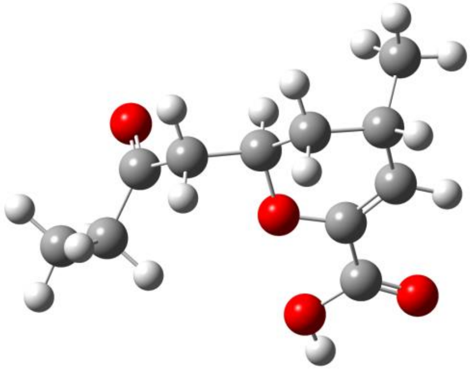

1a

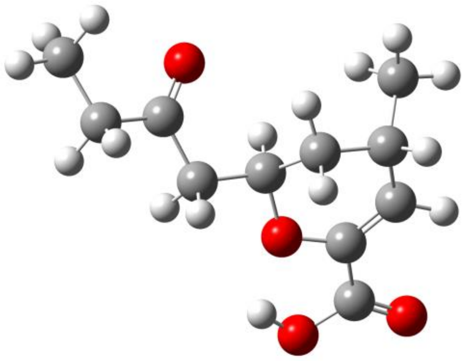

1b

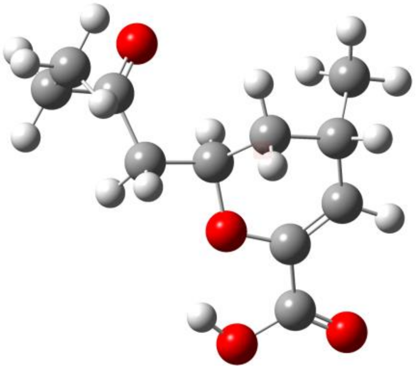

1c

Table S1. Gibbs free energies[a] and equilibrium populations[b] of low-energy conformers of (2*R*, 4*S*)-**1**.

Conformers	In MeOH	
	ΔG	P (%)
**1a**	0	63.69
**1b**	0.44	30.08
**1c**	1.38	6.23

[a]B3LYP/6-31+G(d,p), in kcal/mol. [b]From Δ*G* values at 298.15K

Table S2. Energies of (2*R*, 4*S*)-**1** at B3LYP/6-31+G(d) in gas phase.

Configuration	Conformer	E (Hartree)	E (kcal/mol)	Populations (%)
(2*R*,4*S*)-**1**	**1a**	-729.7325298	-457973.2032	63.69
(2*R*,4*S*)-**1**	**1b**	-729.7318214	-457972.7587	30.08
(2*R*,4*S*)-**1**	**1c**	-729.7303354	-457971.8261	6.23

Table S3. Cartesian coordinates for the low-energy reoptimized MMFF conformers of (2*R*, 4*S*)-**1** at B3LYP/6-311+G(d) level of theory in CH_3OH.

**1a**		Standard Orientation (Ångstroms)			
Center Number	Atomic Number	Atomic Type	X	Y	Z
1	6	0	-0.142657	-1.052268	-0.200086
2	8	0	0.178181	0.333300	-0.402669
3	6	0	1.484097	0.684799	-0.120718
4	6	0	2.487376	-0.203339	-0.009249
5	6	0	2.297984	-1.677472	-0.228310

6	6	0	0.917995	-1.953952	-0.838543
7	6	0	2.503686	-2.436825	1.083137
8	6	0	1.806021	2.126197	0.055488
9	8	0	0.715337	2.906947	-0.074304
10	8	0	2.903009	2.603025	0.292253
11	6	0	-1.509492	-1.299875	-0.834121
12	6	0	-3.133420	0.671869	-0.437823
13	6	0	-2.595514	-0.673778	0.016460
14	6	0	-4.206744	1.213120	0.489436
15	8	0	-2.992499	-1.238783	1.036011
16	1	0	-0.199356	-1.234088	0.881184
17	1	0	3.496639	0.132692	0.221247
18	1	0	3.058510	-2.023940	-0.939361
19	1	0	0.651073	-3.012022	-0.734241
20	1	0	0.968364	-1.737975	-1.914829
21	1	0	1.788626	-2.127608	1.853096
22	1	0	2.386985	-3.514795	0.928138
23	1	0	3.511620	-2.266581	1.478272
24	1	0	1.041982	3.819565	0.060819
25	1	0	-1.730081	-2.371234	-0.904280
26	1	0	-1.565987	-0.882607	-1.845789
27	1	0	-2.302525	1.382262	-0.484449
28	1	0	-3.548387	0.550906	-1.443889
29	1	0	-5.057764	0.526637	0.549385
30	1	0	-3.816441	1.352773	1.502991
31	1	0	-4.571473	2.179244	0.127350

1b		Standard Orientation (Ångstroms)			
Center Number	Atomic Number	Atomic Type	X	Y	Z
1	6	0	-0.131810	-0.910905	-0.100617
2	8	0	0.345501	0.410067	-0.411508
3	6	0	1.693770	0.605892	-0.180058
4	6	0	2.589846	-0.386195	-0.057442
5	6	0	2.216339	-1.834721	-0.197869
6	6	0	0.781227	-1.971982	-0.726508
7	6	0	2.398081	-2.556222	1.138460
8	6	0	2.138568	2.022580	-0.054791
9	8	0	1.058733	2.842979	-0.061916
10	8	0	3.292067	2.403277	0.046876
11	6	0	-1.553264	-1.031841	-0.655138
12	6	0	-3.989601	-0.258201	-0.366426
13	6	0	-2.541314	-0.136938	0.078727
14	6	0	-4.917717	0.667239	0.399354
15	8	0	-2.191026	0.640579	0.965346
16	1	0	-0.155042	-1.021239	0.991583
17	1	0	3.636443	-0.157890	0.135040
18	1	0	2.892937	-2.300158	-0.925532
19	1	0	0.394187	-2.981464	-0.545464
20	1	0	0.802238	-1.824975	-1.815346
21	1	0	1.761392	-2.132782	1.922810
22	1	0	2.150986	-3.618808	1.040883
23	1	0	3.437015	-2.488314	1.480541
24	1	0	0.231017	2.311102	-0.123214

25	1	0	-1.908037	-2.063651	-0.556227
26	1	0	-1.575378	-0.742226	-1.712055
27	1	0	-4.038953	-0.023382	-1.434809
28	1	0	-4.306058	-1.296187	-0.220086
29	1	0	-5.947798	0.549032	0.049390
30	1	0	-4.893544	0.448865	1.472112
31	1	0	-4.628351	1.714621	0.263848

**1c**			Standard Orientation (Ångstroms)		
Center Number	Atomic Number	Atomic Type	X	Y	Z
1	6	0	0.294663	0.704782	-0.122941
2	8	0	-0.432935	-0.506753	-0.392162
3	6	0	-1.793754	-0.425412	-0.164808
4	6	0	-2.477095	0.727100	-0.080919
5	6	0	-1.825862	2.068404	-0.265338
6	6	0	-0.392159	1.902924	-0.789332
7	6	0	-1.861580	2.854773	1.046035
8	6	0	-2.509867	-1.721620	0.001881
9	8	0	-1.613761	-2.739186	0.025896
10	8	0	-3.715803	-1.863405	0.108852
11	6	0	1.712150	0.525967	-0.672614
12	6	0	3.940821	-0.733604	-0.327448
13	6	0	2.501616	-0.522844	0.095226
14	6	0	4.857262	0.263149	0.352763
15	8	0	2.018675	-1.169868	1.024474
16	1	0	0.339683	0.845610	0.965056

17	1	0	-3.548380	0.715680	0.110833
18	1	0	-2.397750	2.633888	-1.011717
19	1	0	0.185926	2.822156	-0.639386
20	1	0	-0.442103	1.726333	-1.872806
21	1	0	-2.893471	3.004230	1.383498
22	1	0	-1.320908	2.339918	1.847592
23	1	0	-1.409702	3.844019	0.916099
24	1	0	-0.697412	-2.383618	-0.047582
25	1	0	2.260214	1.471640	-0.607005
26	1	0	1.674707	0.202622	-1.719268
27	1	0	4.230094	-1.757030	-0.064151
28	1	0	4.010992	-0.651204	-1.416982
29	1	0	4.607864	1.290275	0.068090
30	1	0	4.775002	0.191543	1.442381
31	1	0	5.898671	0.075918	0.073830

Table S4. Tyrosinase inhibition rate (%) of compounds **2–8** at different concentrations (μM).

Compounds	**2**	**3**	**4**	**5**	**6**	**7**	**8**
10 μM	-11.11 %	-2.47 %	-1.85 %	-2.47 %	-6.17 %	0 %	-2.47 %
20 μM	-10.49 %	-3.09 %	-4.94 %	-6.17 %	-6.17 %	0 %	-6.17 %
40 μM	-12.96 %	-2.47 %	-4.94 %	-8.02 %	-5.56 %	0.62 %	-3.70 %
